# Whole brain helical Tomotherapy with integrated boost for brain metastases in patients with malignant melanoma–a randomized trial

**DOI:** 10.1186/1748-717X-8-234

**Published:** 2013-10-10

**Authors:** Henrik Hauswald, Gregor Habl, David Krug, Denise Kehle, Stephanie E Combs, Justo Lorenzo Bermejo, Jürgen Debus, Florian Sterzing

**Affiliations:** 1 Department of Radiation Oncology, University Hospital of Heidelberg, INF 400, 69120, Heidelberg, Germany; 2 Institute of Medical Biometry and Informatics, University of Heidelberg, INF 305, 69120, Heidelberg, Germany

**Keywords:** Malignant melanoma, Brain metastases, Radiotherapy, Tomotherapy, Integrated boost

## Abstract

**Background:**

Patients with malignant melanoma may develop brain metastases during the course of the disease, requiring radiotherapeutic treatment. In patients with 1–3 brain metastases, radiosurgery has been established as a treatment option besides surgery. For patients with 4 or more brain metastases, whole brain radiotherapy is considered the standard treatment. In certain patients with brain metastases, radiation treatment using whole brain helical Tomotherapy with integrated boost and hippocampal-sparing may improve prognosis of these patients.

**Methods/Design:**

The present prospective, randomized two-armed trial aims to exploratory investigate the treatment response to conventional whole brain radiotherapy applying 30 Gy in 10 fractions versus whole brain helical Tomotherapy applying 30 Gy in 10 fractions with an integrated boost of 50 Gy to the brain metastases as well as hippocampal-sparing in patients with brain metastases from malignant melanoma. The main inclusion criteria include magnetic resonance imaging confirmed brain metastases from a histopathologically confirmed malignant melanoma in patients with a minimum age of 18 years. The main exclusion criteria include a previous radiotherapy of the brain and not having recovered from acute high-grade toxicities of prior therapies. The primary endpoint is treatment-related toxicity. Secondary endpoints include imaging response, local and loco-regional progression-free survival, overall survival and quality of life.

**Trial registration:**

http://www.drks.de Trial ID: DRKS00005127

## Background

Malignant melanomas (MM) account for 4.3% of all cancers in Germany in females and 3.2% in males, respectively [1]. In the last 3 decades, the age standardized incidence rates in Germany tripled [1]. In general, survival in these patients is limited, and reported to be around 4 to 5 months while MM cause 1% of all cancer deaths [1-3]. With respect to tumor stage, about 10–13% of patients initially presenting with AJCC Stage III disease are at risk for brain metastases (BM) [4-7], and 18–46% of stage IV patients will develop CNS involvement [8]. Factors associated with development of BM are male gender, mucosal or head and neck primaries, as well as deep or ulcerated lesions [9].

Diagnosis of BM from MM is commonly associated with a poor prognosis. Surgery is evaluated, however lesions can be too small for safe neurosurgical resection, or multiple lesions are diagnosed arguing against a neurosurgical intervention; this situation holds true for the majority of MM patients with BM [10].

For multiple metastases standard of care is whole brain radiotherapy (WBRT) delivered as 40 Gy in 2 Gy fractions, or 30 Gy in 3 Gy fractions, leading to modest palliation with median survival times of 3–5 months, and 1-year survival rates of about 13% [11-18]. Our own data showed median overall survival of 3.5 months and a 12-months overall survival rate of 16.5% [19]. Prognostic factors influencing survival are predominantly the radiation therapy oncology group (RTOG) recursive partitioning analysis (RPA) class and Karnofsky performance score (KPS) [12,20,21]. In patients with local treatment, such as neurosurgical resection or stereotactic radiosurgery (SRS) which is commonly followed by WBRT, progression-free survival, and in some cases, overall survival can be improved [22,23].

Due to the limited efficacy of WBRT and the advent of high precision radiosurgery techniques, local radiotherapeutic treatment with SRS has been established for subgroups of patients with BM, especially with 1–3 lesions. It has been shown that in these patients WBRT plus surgery or SRS leads to an increase of loco-regional control compared to local treatments alone, but overall survival is unaltered [23]. However, in this analysis, several tumor types with high numbers of breast or lung tumors were included. For MM patients, the clinical efficacy or WBRT is known to be limited, and therefore, these patients are even often excluded from trials evaluating efficacy of treatment for BM. Therefore, in patients with limited intracranial disease (1–3 metastases), SRS can be chosen as an effective regimen. Several groups have reported high efficacy of SRS for patients with BM from MM: Seung and colleagues treated 140 lesions in 46 patients, with progression-free rates of 86% and 76% at 6 and 12 months, respectively [24,25]; in this group about 50% of the patients had been treated with SRS alone. Patients with multiple lesions showed a reduced outcome compared to patients with solitary metastases. Grob and co-workers published local control rates of 98% at 3 months for 56 metastases in 35 patients treated with SRS only and without additional WBRT. Median survival was longer in patients with single lesions, for example 7.5 months versus 4 months [26]. Our own data published previously were based on 64 patients treated for 122 lesions: in this group, the local control was 81% at 12 months, and the median survival after treatment 10.6 months [27].

Sterzing et al. could show the possibilities of helical Tomotherapy in the setting of a re-irradiation for multiple metastases with a whole brain radiation including a multifocal simultaneous integrated boost [28]. Nevertheless it emphasizes the possibilities of Tomotherapy to overcome limitations of classical WBRT and allows selective tailor-made dose escalation [28]. Furthermore, Marsh et al. reported on the feasibility of neural stem cell and hippocampus region sparing during WBRT by using helical Tomotherapy and a “how-to” on helical Tomotherapy for hippocampal-sparing WBRT was published by Gondi et al. [29,30].

In the present randomized and prospective two-armed trial, the toxicity of the radiotherapeutic treatment in patients with BM from MM treated with either WBRT or whole brain helical Tomotherapy (WBHT) with integrated boost and hippocampal-sparing will be investigated.

## Methods and design

### Study design

**Figure 1:**
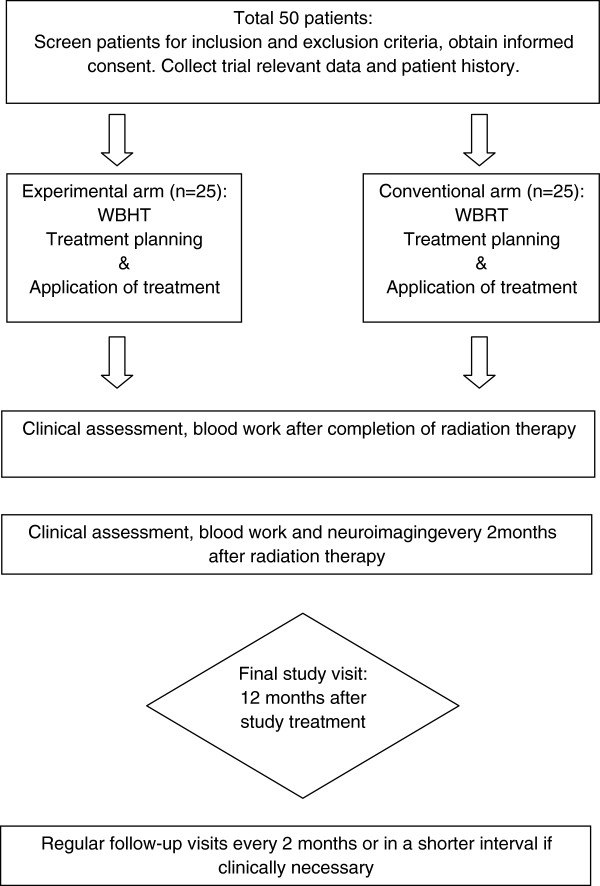
Flow chart of the study.

The BRAIN-RT study is a prospective, randomized two-arm exploratory study designed to generate information on the safety and treatment response of WBHT with integrated boost and hippocampal-sparing for BM from MM. The flow chart is found in Figure 1. After thorough information patients have to provide a written informed consent before being enrolled into this study. Following the baseline assessments, the patients are randomized to either experimental WBHT or conventional WBRT:

Experimental arm 1: WBHT applying 30 Gy in 10 fractions to the whole brain with an integrated boost of 50 Gy in 10 fractions to the BM. Furthermore, hippocampal-sparing as low as reasonable achievable should be aspired.

Conventional arm 2: WBRT applying 30 Gy in 10 fractions to the whole brain.

### Endpoints

The primary endpoint of this study is toxicity. The secondary endpoints are imaging response, local and loco-regional progression-free survival, overall survival and quality of life (QoL). The toxicity will be quantified according to the common toxicity criteria (CTC) Version 4. The imaging response will be evaluated according to the response evaluation criteria in solid tumors (RECIST 1.1). QoL will be measured using the European Organisation for Research and Treatment of Cancer (EORTC)-QoL-Q C30 and brain module BN-20. Furthermore, translational investigations for immune monitoring will be performed. The endpoints will be accessed after enclosure of the participants, at the end of radiation therapy and every two months thereafter up to a total of 12 months.

### Patient selection criteria

The BRAIN-RT study encloses patients with magnetic resonance imaging (MRI) confirmed brain metastases (n > 1) from a histopathologically confirmed malignant melanoma. All patients fulfilling the inclusion and exclusion criteria will be informed about the possibility to participate in the study. Registration for the study must be performed prior to beginning of RT. The detailed in- and exclusion criteria are listed in Table 1.

**Table 1:** In- and exclusion criteria of the study

**Inclusion criteria**	
Patients meeting all of the following criteria will be considered for admission to the trial:	
•	histologically confirmed malignant melanoma (MM)
•	MR-imaging confirmed >1 cerebral metastases (in case of resection, >1 remaining metastases)
•	age ≥ 18 years of age
•	Karnofsky Performance Score ≥60
•	For women with childbearing potential, (and men) adequate contraception.
•	Ability of subject to understand character and individual consequences of the clinical trial
•	Written informed consent (must be available before enrolment in the trial)
**Exclusion criteria**	
Patients presenting with any of the following criteria will not be included in the trial:	
•	refusal of the patients to take part in the study
•	previous radiotherapy of the brain
•	Patients who have not yet recovered from acute high-grade toxicities of prior therapies
•	Known carcinoma < 5 years ago (excluding Carcinoma in situ of the cervix, basal cell carcinoma, squamous cell carcinoma of the skin) requiring immediate treatment interfering with study therapy
•	Pregnant or lactating women
•	Participation in another clinical study or observation period of competing trials, respectively
•	MRI contraindication (i.e. cardiac pacemaker, implanted defibrillator, certain cardiac valve replacements, certain metal implants)

### Ethical aspects

Approval by the ethics committee of the University of Heidelberg (S-327/2012) and the Federal Office of Radiation Protection (BfS) (Z 5-22461/2-2013-001) has been obtained. The trial is registered at http://www.drks.de (trial ID: DRKS00005127).

### Radiation therapy/treatment planning and dose prescription

Experimental arm 1: Patients will be immobilized using an individually manufactured head mask. For treatment planning, CT with and without contrast-enhancement as well as MR-imaging will be performed for optimal target definition. Organs at risk such as the brain stem, optic nerves, chiasm, hippocampus and spinal cord will be contoured. Dose constraints of normal tissue will be respected according to the quantitative analyses of normal tissue effects in the clinic (QUANTEC) reports [31]. The clinical target volume (CTV) includes the whole brain. PTV whole brain = CTV + 5 mm. The Gross Tumor Volume (GTV) for the integrated boost will be defined as the contrast-enhancing lesions on T1-weighted MR-imaging. The Planning Target Volume (PTV) will include a safety margin of up to 1–2 mm. For low infratentorial lesions, the CTV may include the whole brain down to the second cervical vertebra.

WBHT will be applied in 10 fractions with median single doses of 3 Gy to the whole brain. The integrated boost will be applied by median single doses of 5 Gy.

WBHT 10 × 3 Gy: The QUANTEC recommendations are adhered. Hippocampus: ALARA.

Integrated boost 10 × 5 Gy: Brain stem: < 73% (=biological 53,8 Gy @ α/β 2,1 Gy and reference dose 1,8 Gy), Optic nerve/chiasm: <76% (=biological 53,8 Gy @ α/β 3,0 Gy and reference dose 1,8 Gy), Hippocampus: ALARA, Spinal cord: <66% (=biological 46,0 Gy @ α/β 2,0 Gy and reference dose 1,8 Gy).

The target volume definition for WBHT is performed using the following software: Nucletron Masterplan, Siemens Dosimetrist and Oncologist. Treatment planning is performed using the Tomotherapy planning software. Prior to WBHT, patient positioning will be evaluated using a megavoltage-CT. Treatment will be delivered using 6 MeV photon beams.

Conventional arm 2: Patients will be immobilized using an individually manufactured head mask. For treatment planning, CT without contrast for a virtual simulation will be performed. The CTV includes the whole brain. WBRT will be delivered by opposed lateral 6 MeV photon beams. For low infratentorial lesions, the CTV may include the whole brain down to the second cervical vertebra. WBRT will be applied in 10 fractions with single doses of 3 Gy to the whole brain.

### Statistical considerations

#### Study hypothesis and sample size

This exploratory study is designed to generate preliminary information on the safety and treatment response of whole brain helical Tomotherapy with integrated boost and hippocampal-sparing for BM from MM. The intention-to-treat population consists of all patients who signed an informed consent and met the inclusion criteria. Each study-arm will include 25 patients. The planned sample size relies on logistic considerations based on the expected annual recruitment of patients affected by MM with multiple BM.

#### Investigated samples/population

The primary analyses will be based on the complete set of patients classified according to the intention-to-treat principle (International Conference on Harmonisation, 1999). This data set includes all patients who were treated at least once. The per-protocol set includes all patients from the complete set without major protocol deviations. The analysis of toxicity and safety will be conducted based on all randomized patients who were treated at least once.

### Statistical methods

The primary endpoint is toxicity. Secondary endpoints are imaging response, local and loco-regional progression-free survival, overall survival and quality of life. Toxicity will be quantified by absolute frequencies and percentages of side effects according to CTC adverse event criteria version 4.

Imaging response will be evaluated according to the RECIST criteria and represented by percentages. Kaplan-Meier curves will be plotted and Cox-regression analyses will be conducted to investigate possible factors influencing survival. QoL will be measured with the EORTC-QoL-Q C30 (http://groups.eortc.be/qol/eortc-qlq-c30) and its brain module BN-20, the results will be summarized by median, minimum and maximum scores, and represented by box plots.

## Discussion

The standardized incidence rates of malignant melanoma tripled in the last decades [1], therefore the management of malignant melanoma becomes more and more important for the daily clinical routine. When looking back to the stage dependant incidence rate, about 10% of patients initially presenting with AJCC Stage III disease are at risk for [4-7] and up to 46% of stage IV patients will develop BM [8]. Due to the relatively short survival time after initial diagnosis of multiple BM as well as low complete remission rates to WBRT [19], management of BM remains a major challenge in the management of this disease. In patients with only few and easily accessible BM surgical resection is standard treatment, a resection of multiple BM is normally not indicated. Furthermore, systemic therapies do not have proven equivalent effectiveness up to date [32]. With the development and clinical implementation of new radiation therapy techniques as Tomotherapy or other intensity-modulated arc techniques, especially if using smaller collimation systems, the application of an integrated boost to BM seems feasible [28] and might promise an increased tumor response resulting in higher remission rates while reducing treatment-related side effects by hippocampal-sparing at the same time [30]. A recent Australian report on 30 patients treated with volumetric modulated arc therapy for BM from mainly MM concluded that treatment was feasible and yielded comparable survival times and side effects compared to standard radiosurgery with or without WBRT [33].

Even though helical Tomotherapy promises excellent homogeneity and conformality in the setting of an integrated boost for BM [28], this technique still has its limitations when compared to a classical radiosurgery: in a plan comparison on radiosurgery versus helical Tomotherapy for arterio-venous malformations, all helical Tomotherapy plans had a higher high-dose brain exposure while only the regular helical Tomotherapy delivery using 0.6 cm field width showed a better low-dose exposure [34]. On the other hand since application is planned as an integrated boost to whole brain radiotherapy, the high-dose exposure should play a minor role. Furthermore, with the implementation of dynamic jaws and dynamic couch the total treatment time compared to regular delivery times as well as dose penumbra and consecutively the integral dose will be reduced [35].

Further aspects are the side effects caused by the integral dose to the whole brain. Unfortunately, there is only limited data on the tolerance dose of the whole brain, most publications available report on tolerance doses for partial brain irradiations. According to the QUANTEC reports, the risk for radiation induced necrosis of the brain is below 3% in case of less than 60 Gy/2 Gy and between 5% in case of a biologically equivalent dose (BED) of 120 Gy (72 Gy/2 Gy) and 10% in case of a BED of 150 Gy (90Gy / 2 Gy) and might increase with hypofractionation. According to our BED calculation one should aspire an integral dose to the whole brain below 65% of the integrated boost dose, corresponding to 44.9 Gy in 1.8 Gy fractions using an α/β of 2.0 Gy and thereby staying below 2.5% risk for radiation necrosis as described in the RTOG low-grade glioma trial by Shaw et al. [36].

Therefore, this randomized, prospective two-armed phase I-II trial is primarily aimed at the safety by evaluation of treatment related toxicity. Secondary p are the imaging response, local and loco-regional tumor control, overall survival and quality of life in patients treated with whole brain helical Tomotherapy with integrated boost and hippocampal-sparing for brain metastases from malignant melanoma. We assume that the treatment results might be improved by additional dose escalation. In case of promising results, the information generated in the present trial will be used to plan a confirmatory study.

## Competing interests

The authors declare that they have no competing interests.

## Authors’ contributions

HH, FS, SC and JD planned the study. HH, FS, SC, JD, GH, DKr and DKe are responsible for patient recruitment. FS, SC, GH, JD and HH perform planning and radiation therapy. JLB performs biometric and statistical analysis. Medical care and follow up is provided by HH, FS, SC, JD, DKr, GH and DKe. HH, SC and FS drafted the manuscript. JD revised the manuscript critically for important intellectual content. All authors have read and approved the final manuscript.
